# 5-HT_2A_ and 5-HT_2C_ receptors as hypothalamic targets of developmental programming in male rats

**DOI:** 10.1242/dmm.023903

**Published:** 2016-04-01

**Authors:** Malgorzata S. Martin-Gronert, Claire J. Stocker, Edward T. Wargent, Roselle L. Cripps, Alastair S. Garfield, Zorica Jovanovic, Giuseppe D'Agostino, Giles S. H. Yeo, Michael A. Cawthorne, Jonathan R. S. Arch, Lora K. Heisler, Susan E. Ozanne

**Affiliations:** 1 University of Cambridge, Metabolic Research Laboratories and MRC Metabolic Diseases Unit, Wellcome Trust-MRC Institute of Metabolic Science, Addenbrooke's Hospital, Cambridge CB2 0QQ, UK; 2 Clore Laboratory, Buckingham Institute for Translational Medicine, University of Buckingham, Hunter Street, Buckingham MK18 1EG, UK; 3 Department of Pharmacology, University of Cambridge, Cambridge CB2 1PD, UK

**Keywords:** Serotonin, Developmental programming, Hypothalamus, Low birth weight, Maternal diet, Protein restriction

## Abstract

Although obesity is a global epidemic, the physiological mechanisms involved are not well understood. Recent advances reveal that susceptibility to obesity can be programmed by maternal and neonatal nutrition. Specifically, a maternal low-protein diet during pregnancy causes decreased intrauterine growth, rapid postnatal catch-up growth and an increased risk for diet-induced obesity. Given that the synthesis of the neurotransmitter 5-hydroxytryptamine (5-HT) is nutritionally regulated and 5-HT is a trophic factor, we hypothesised that maternal diet influences fetal 5-HT exposure, which then influences development of the central appetite network and the subsequent efficacy of 5-HT to control energy balance in later life. Consistent with our hypothesis, pregnant rats fed a low-protein diet exhibited elevated serum levels of 5-HT, which was also evident in the placenta and fetal brains at embryonic day 16.5. This increase was associated with reduced levels of 5-HT_2C_R, the primary 5-HT receptor influencing appetite, in the fetal, neonatal and adult hypothalamus. As expected, a reduction of 5-HT_2C_R was associated with impaired sensitivity to 5-HT-mediated appetite suppression in adulthood. 5-HT primarily achieves effects on appetite by 5-HT_2C_R stimulation of pro-opiomelanocortin (POMC) peptides within the arcuate nucleus of the hypothalamus (ARC). We show that 5-HT_2A_Rs are also anatomically positioned to influence the activity of ARC POMC neurons and that mRNA encoding 5-HT_2A_R is increased in the hypothalamus of *in utero* growth-restricted offspring that underwent rapid postnatal catch-up growth. Furthermore, these animals at 3 months of age are more sensitive to appetite suppression induced by 5-HT_2A_R agonists. These findings not only reveal a 5-HT-mediated mechanism underlying the programming of susceptibility to obesity, but also provide a promising means to correct it, by treatment with a 5-HT_2A_R agonist.

## Abstract (precis)

**Summary:** Maternal protein restriction increases fetal brain 5-HT and might contribute to changes in production and function of hypothalamic 5-HT_2C_ and 5-HT_2A_ receptors in the offspring later in life.

## INTRODUCTION

Nutrition during critical periods of development in early life can exert profound, long-term effects on susceptibility to obesity. For example, men exposed to the Dutch ‘Hunger Winter’ *in utero* during early gestation had an increased risk of developing obesity as adults, whereas obesity rates were reduced amongst those exposed to famine during the last trimester of gestation and in early postnatal life (Ravelli et al., 1976). The early postnatal diet is also important. In randomised trials, full-term infants with low birth weight fed a growth-promoting nutrient-enriched formula had a higher fat mass at the age of 5-8 years than those fed standard formula (Singhal et al., 2010). Studies in rodents, showing that low birth weight followed by rapid postnatal growth is associated with increased adiposity, support findings in humans (Plagemann et al., 1992; Cottrell et al., 2011; Berends et al., 2013). Despite these robust associations, the molecular mechanisms mediating the interaction between early nutrition and obesity risk are still largely unknown.

It is widely acknowledged that the hypothalamus, which in humans develops primarily prenatally but in rodents develops postnatally, plays an important role in the programming of body mass (Grove et al., 2005; Horvath and Bruning, 2006; Glavas et al., 2007). To date, major efforts have been directed at understanding the roles of leptin and insulin in this process (Bouret, 2010; Yura et al., 2005; Steculorum and Bouret, 2011). However, studies in our laboratory using leptin-deficient *ob*/*ob* mice demonstrated that leptin-independent mechanisms are likely to also programme body mass (Cottrell et al., 2011).

The importance of the 5-hydroxytryptamine (5-HT; serotonin) system in the control of food intake and body mass has been recognised for many years and alterations in central serotonergic activity have been observed in obese humans, non-human primates and rodents (Mori et al., 1999; De Fanti et al., 2001; Sullivan et al., 2010). 5-HT is a potent anorectic signal that influences food intake in the mature brain by acting predominantly via 5-HT_2C_R to regulate the key energy balance mediator, pro-opiomelanocortin (POMC), within the arcuate nucleus of the hypothalamus (ARC; Doslikova et al., 2013; Burke et al., 2014). Indeed, disruption of the 5-HT_2C_R gene (*Htr2c*) specifically expressed on POMC neurons programmes hyperphagia and obesity when animals are fed a high-fat diet (Berglund et al., 2013). In addition to playing an important role in energy balance and body mass, 5-HT acts as a trophic factor during fetal brain development in rodents, regulating cell migration, proliferation, maturation and axonal growth (Whitaker-Azmitia et al., 1996; Gaspar et al., 2003). The long-term effects of disturbances in the 5-HT system during early life on obesity risk are, however, not fully defined.

Here, we used an unbiased genome-wide profiling approach to identify differentially expressed targets in the hypothalamus of intrauterine protein-restricted rats that underwent postnatal catch-up growth (recuperated animals) in an effort to elucidate the molecular mechanisms that mediate the effects of early nutrition on energy balance.

## RESULTS

### Growth trajectories, body and brain mass

At birth, offspring of mothers fed a low protein diet during pregnancy were significantly smaller than the control pups (*P*<0.001) (Table 1A). The body mass of the recuperated pups caught up with that of control pups by postnatal day (P)14 and they weighed the same as controls at weaning (Table 1A). Fractional growth rate did not differ between recuperated and control pups between birth and P7 (0.16±0.1 vs 0.17±0.01); however, recuperated offspring had significantly higher fractional growth rate between birth and P14 and P21 when compared with controls (P14: 0.31±0.01 vs 0.24±0.01, *P*<0.001; P21: 0.38±0.01 vs 0.29±0.01, *P*<0.001). At 3 months of age, body masses were similar between recuperated and control offspring. Brain masses were similar between the experimental animals and their age-matched controls at both P22 and 3 months of age, in both absolute terms and relative to body mass (Table 1B). These findings demonstrate that maternal low-protein diet decreases *in utero* growth of the offspring so that they have a lower birth weight. These animals then undergo ‘catch up’ growth such that by P22, they have the same body mass as the control offspring.

**Table 1.**
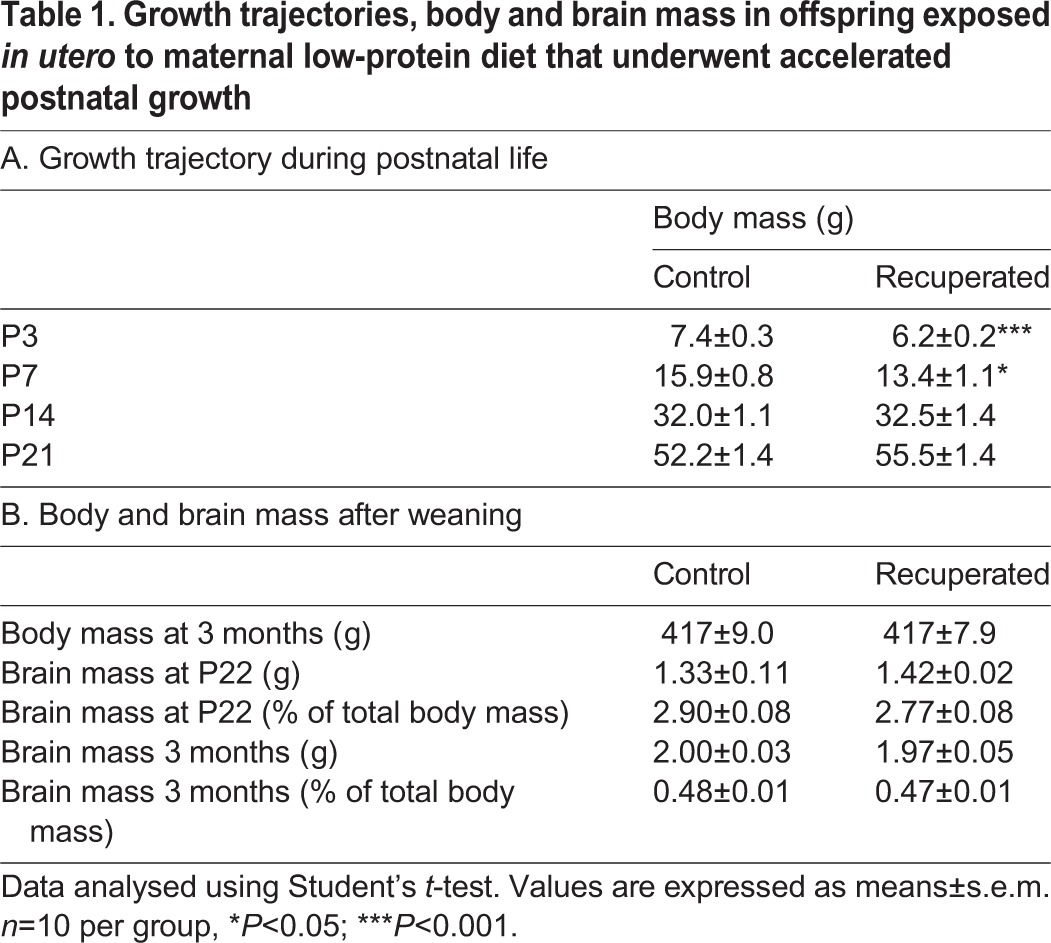
**Growth trajectories, body and brain mass in offspring exposed *in utero* to maternal low-protein diet that underwent accelerated postnatal growth**

### Levels of 5-HT and tryptophan in mothers and offspring

#### Maternal, placental and amniotic fluid 5-HT and tryptophan levels

**Fig. 1.**
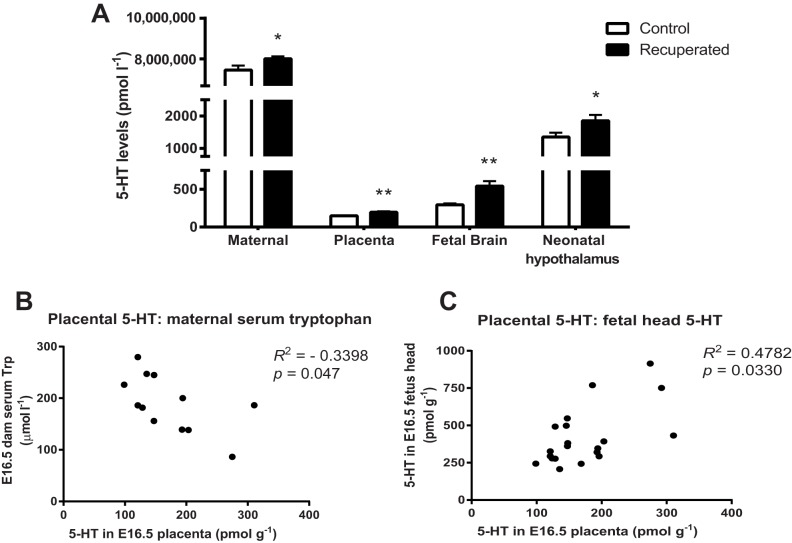
**Effects of maternal nutrition on 5-HT levels in low-protein and control pregnancies, and neonatal pups**. (A) 5-HT levels in maternal serum, the placenta, fetal brain (all analysed at E16.5) and neonatal hypothalamus. Data analysed using unpaired Student's *t*-test and presented as mean percentage of control; for maternal serum 5-HT, *n*=6; placenta 5-HT, *n*=16; fetal brain 5-HT, *n*=10; neonatal hypothalamus, *n*=12 per group. (B,C) Correlation between maternal, placental and fetal 5-HT levels. (B) Correlation between placental 5-HT and maternal serum tryptophan, analysed using Pearson correlation coefficient because data were normally distributed; *n*=6 per group. (C) Correlation between placental 5-HT and fetal head 5-HT analysed using Spearman correlation coefficient because data were not normally distributed. *n*=10 per group; *n* represents number of litters. *P<0.05; **P<0.01.

Tryptophan is an essential amino acid and is required for 5-HT synthesis. A low-protein diet would therefore be expected to yield low plasma tryptophan levels. We analysed the levels of 5-HT and tryptophan in the dams, placenta and amniotic fluid to investigate whether 5-HT might be a programming factor that mediates changes in appetite and susceptibility to diet-induced obesity in recuperated animals. As expected, at embryonic day (E)16.5, we observed reduced levels of tryptophan in the serum of dams fed a low-protein diet (‘low-protein’) compared with control dams (158±17 mmol l^−1^ vs 220±20 mmol l^−1^, *P*<0.05). Paradoxically, the serum 5-HT level was raised in low-protein dams at this time point (*P*<0.05; Fig. 1A). A similar pattern was observed for tryptophan and 5-HT concentrations in the amniotic fluid at E16.5, with tryptophan levels reduced in the amniotic fluid from low protein pregnancies (26.3±3.2 mmol l^−1^ vs 40.6±3.4 mmol l^−1^, *P*<0.01, *n*=10-11 per group), whereas 5-HT levels were raised (65.0±6.6 nmol l^−1^ vs 48.2±3.9 nmol l^−1^, *P*<0.05, *n*=10-11 per group). 5-HT levels were also significantly increased in E16.5 placentas from low-protein pregnancies when compared with control pregnancies (*P*<0.01; Fig. 1A). Furthermore, we observed a negative correlation between placental 5-HT level and maternal serum tryptophan (*P*<0.05; Fig. 1B). There was a positive correlation between placental 5-HT and fetal brain 5-HT levels (Fig. 1C). These findings reveal that in response to significantly reduced dietary tryptophan, 5-HT is overproduced in the mother and in the intrauterine environment in which the fetus grows and develops.

#### Fetal, neonatal, weaning and adult tryptophan and 5-HT levels

Likewise, in the neonatal brain, a decrease in tryptophan levels (67.9±13.6 mmol l^−1^ vs 140±14.7 mmol l^−1^, *P*<0.01) was associated with an increase in 5-HT levels in the hypothalami of recuperated pups at birth (*P*<0.01; Fig. 1A). 5-HT levels were also increased in the brains of E16.5 fetuses from low-protein pregnancies (Fig. 1A). By weaning as well as at 3 months, hypothalamic 5-HT levels were normalised to control levels (P21: recuperated, 1347±110 mmol l^−1^ vs control, 1659±131 pmol g^−1^; *n*=12 per group; 3 months: recuperated, 1414±154 mmol l^−1^ vs control, 1354±85 pmol g^−1^; *n*=10 per group). These data indicate that the overproduction of 5-HT in response to a maternal low-protein diet is normalised by weaning.

### Effect of D-fenfluramine on food intake

**Fig. 2.**
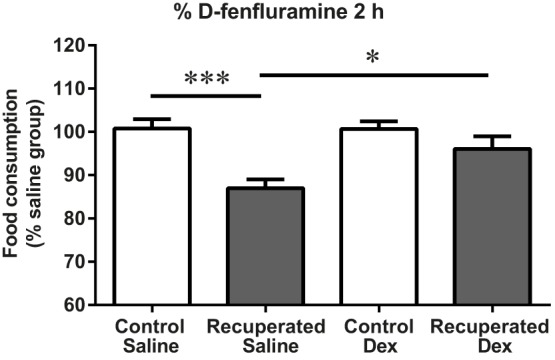
**Effects of maternal protein restriction on food intake in 3-month-old offspring following central administration of D-fenfluramine (Dex).** Food consumption during the 2 h following lights out expressed as a percentage of the saline dosed group. Difference between the groups analysed by one-way ANOVA to saline for control and recuperated offspring. *n*=6 per group with *n* representing number of litters. The following numbers of animals were used for each experimental group: control saline, *n*=18 rats; control Dex, *n*=20; recuperated saline; *n*=17; recuperated Dex, *n*=20 rats. D-fenfluramine was administered at 250 nmoles in 2.5 μl of saline. *P<0.05 for control Dex versus recuperated Dex. ***P<0.001 for control saline versus control Dex.

We next assessed the significance of maternal diet-stimulated increase in 5-HT during early development of the offspring for the function of the adult 5-HT system. D-fenfluramine is a pharmacological probe of the endogenous 5-HT system that works by stimulating endogenous 5-HT release and blocking its reuptake. D-fenfluramine was prescribed until the 1990s for human obesity treatment because increasing endogenous 5-HT bioavailability reduces appetite and body weight (Lam et al., 2010; Marston et al., 2011). We administered D-fenfluramine into the third ventricle of rats at 3 months and observed the expected reduction in food intake in controls (Fig. 2). However, recuperated offspring had impaired sensitivity to the 5-HT-stimulated (D-fenfluramine) food intake reduction (*P*<0.05) (Fig. 2). These results show that a low-protein maternal diet followed by accelerated postnatal growth diminishes 5-HT-mediated control of food intake in the adult offspring. Given the strong inverse correlation between 5-HT and appetite, these findings led us to hypothesise that a deregulated 5-HT system might contribute to the programmed increased food intake in these offspring when exposed to an obesogenic diet.

### Fetal, neonatal and postnatal expression of *Htr2c* in the hypothalamus

**Fig. 3.**
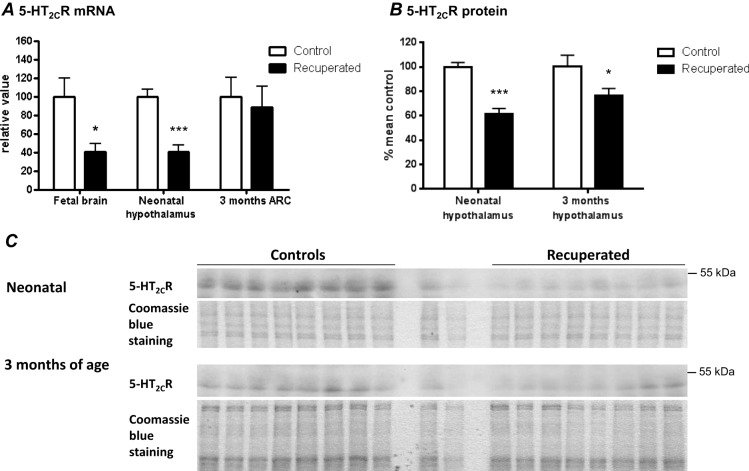
**Effects of maternal protein restriction on central *Htr2c* mRNA and 5-HT_2C_R protein expression in the offspring.** (A) *Htr2c* mRNA levels as measured using qRT-PCR. Gene expression data was normalised to *Ppia*. (B) Analysis of 5-HT_2C_R protein levels from western blot data. (C) Western blots. 20 mg and 10 mg of pooled samples were loaded in the middle of the gels to ensure the linearity of the signal. Data were analysed using unpaired Student's *t*-test and presented as mean±s.e.m. for RNA data and percentages relative to control for protein data; *n*=8 per group, *n* represents number of litters. *P<0.05; ***P<0.001.

5-HT is a neurotransmitter that communicates appetite-related signals primarily through the 5-HT_2C_R within the ARC. If the effects of 5-HT on appetite are diminished in recuperated rats, this suggests a perturbation in signalling at the 5-HT_2C_R. We next probed the consequence of elevated 5-HT during development on the expression of the 5-HT_2C_R. Analysis of *Htr2c* mRNA expression in E16.5 fetal brain showed significantly reduced levels in the whole heads of fetuses from low-protein pregnancies (*P*<0.05; Fig. 3A). At birth, the *Htr2c* transcript level was still reduced in the hypothalamus of the growth-restricted pups (*P*<0.001; Fig. 3A) as was 5-HT_2C_R protein (*P*<0.001; Fig. 3B,C). However, by 3 months, no significant differences in mRNA levels were observed between the experimental groups (Fig. 3A). Protein expression was, however, still significantly reduced in the hypothalamus of recuperated animals at 3 months (*P*<0.05; Fig. 3B,C). Given that perturbations of ARC 5-HT_2C_R function increases appetite and body mass (Berglund et al., 2013), these results reveal a potential mechanism through which rapid postnatal growth is achieved in recuperated rats, which could also contribute to increased susceptibility to diet-induced obesity in these animals.

### Microarray analysis of ARC from control and recuperated 3-month-old offspring

5-HT is a trophic factor during fetal brain development influencing both 5-HT circuits and the anatomical organisation of other systems (Whitaker-Azmitia et al., 1996; Gaspar et al., 2003). The ARC is a crucial regulator of energy balance. Having demonstrated that maternal diet induces changes in offspring 5-HT and disrupts the functioning of the 5-HT appetite system, we next probed whether 5-HT disrupts other ARC genes and circuits regulating appetite. To achieve this, we performed laser-capture microdissection (LCM) of the ARC of 3-month-old control and recuperated rats and subjected samples to microarray analysis.

#### Validation of laser-capture microdissection

**Fig. 4.**
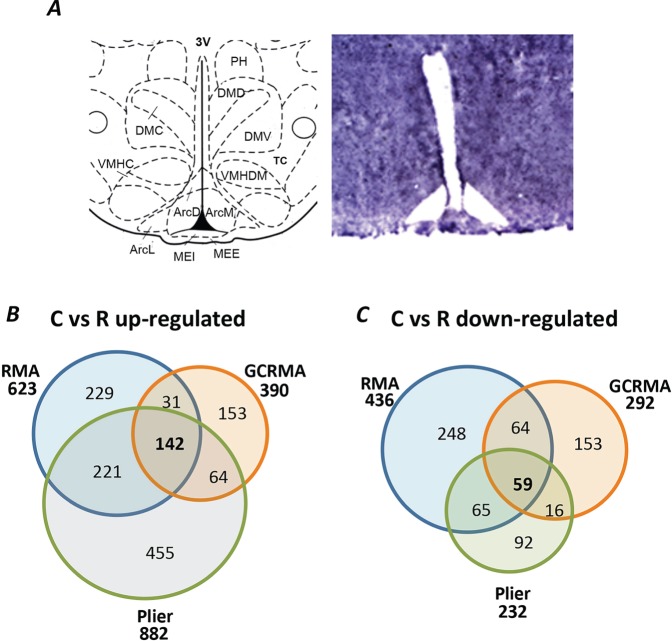
**Laser-capture microdissection (LCM) of the hypothalamic ARC of 3-month-old rats and microarray analysis results.** (A) LCM of the ARC (−4.52 to −2.30 mm relative to Bregma). Brain atlas image was taken from Paxinos and Watson (1998). 3V, 3rd ventricle; ArcD, arcuate nucleus, dorsal part; ArcL, arcuate nucleus, lateral part; ArcM, arcuate nucleus, medial part; DMC, dorsomedial nucleus, compact part; DMD, dorsomedial nucleus, dorsal part; DMV, dorsomedial nucleus, ventral part; MEI, internal layer; MEE, median eminence, external layer; Pe, periventricular hypothalamic nucleus; PH, posterior hypothalamic nucleus; PeF, perifornical nucleus; TC, tuber cinereum area; VMHC, ventromedial nucleus, central part; VMHDM, ventromedial nucleus, dorsomedial part; VMHVL, ventromedial nucleus, ventrolateral part. (B,C) Venn diagrams showing the effects of maternal protein restriction followed by catch-up growth on the expression of genes in the ARC of male 3-month-old offspring, according to three different, robust, analyses: GeneChip operating software (GCOS), GeneChip robust multi-array averaging (GC-RMA) and robust multi-array averaging (RMA). Genes were considered to be up- or downregulated if the 1.3-fold threshold was reached and *P*<0.05. (B) Upregulated genes and (C) downregulated genes in the recuperated animals when compared with controls. The sizes of circles and numbers in parentheses indicate the number of genes as identified by either the GCOS, RMA or GC-RMA algorithms. For microarray analysis *n*=6 chips per group, *n* represents number of litters.

To ensure the specificity of the ARC dissection within the LCM material (Fig. 4), we analysed the expression of two genes that are not expressed in the ARC but are expressed in the neighbouring hypothalamic nuclei. Neither *Brn1* (also known as *Pou3f3*), which is specific to the paraventricular nucleus (PVN) nor *Sf-1* (also known as *Nr5a1*), which is found in the ventromedial nucleus of the hypothalamus (VMN), were detected (data not shown). In addition, we examined the expression of four key genes involved in the regulation of energy balance in the ARC: *Pomc*, *Agrp*, *Cartpt* and *Npy*. We confirmed that all of these genes were highly expressed in the dissected material using Taqman RT-PCR (data not shown).

#### Analysis of microarrays using three different algorithms and pathway analysis

Following global transcriptional profiling, we analysed the data using three different algorithms: (1) GeneChip operating software (GCOS), (2) robust multi-array averaging (RMA) and (3) GeneChip robust multi-array averaging (GCRMA), to ensure maximum stringency and to reduce the number of false positives. The combined analysis, using a 1.3-fold cut-off threshold and *P*<0.05, revealed that out of 31,099 genes analysed, expression of 15,951 genes was detected in the ARC. Of these, 142 genes were upregulated in the ARC of the recuperated animals, whereas 59 genes were downregulated (Fig. 4B,C, respectively). The top 25 up- and downregulated genes in recuperated offspring are presented in Tables 2 and 3. *Htr2a*, which encodes 5-HT_2A_R, was the gene with the highest (2.55-fold) increase. We further analysed the microarray data using Ingenuity Pathway analysis software to detect groups of functionally related genes. The top three gene functions affected by early nutrition in 3-month-old male recuperated rats were cell cycle, connective tissue development and function, cellular growth and proliferation (Table 4).

**Table 2.**
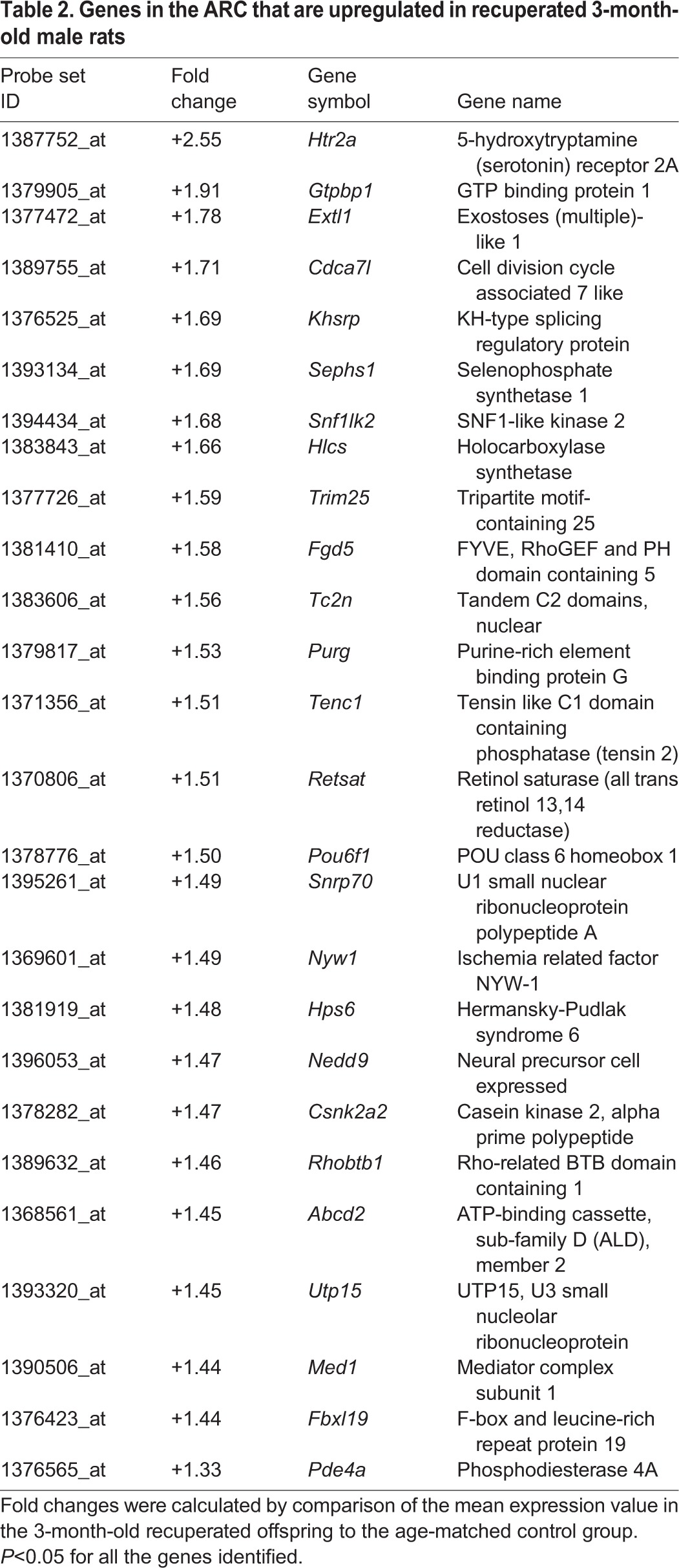
**Genes in the ARC that are upregulated in recuperated 3-month-old male rats**

**Table 3.**
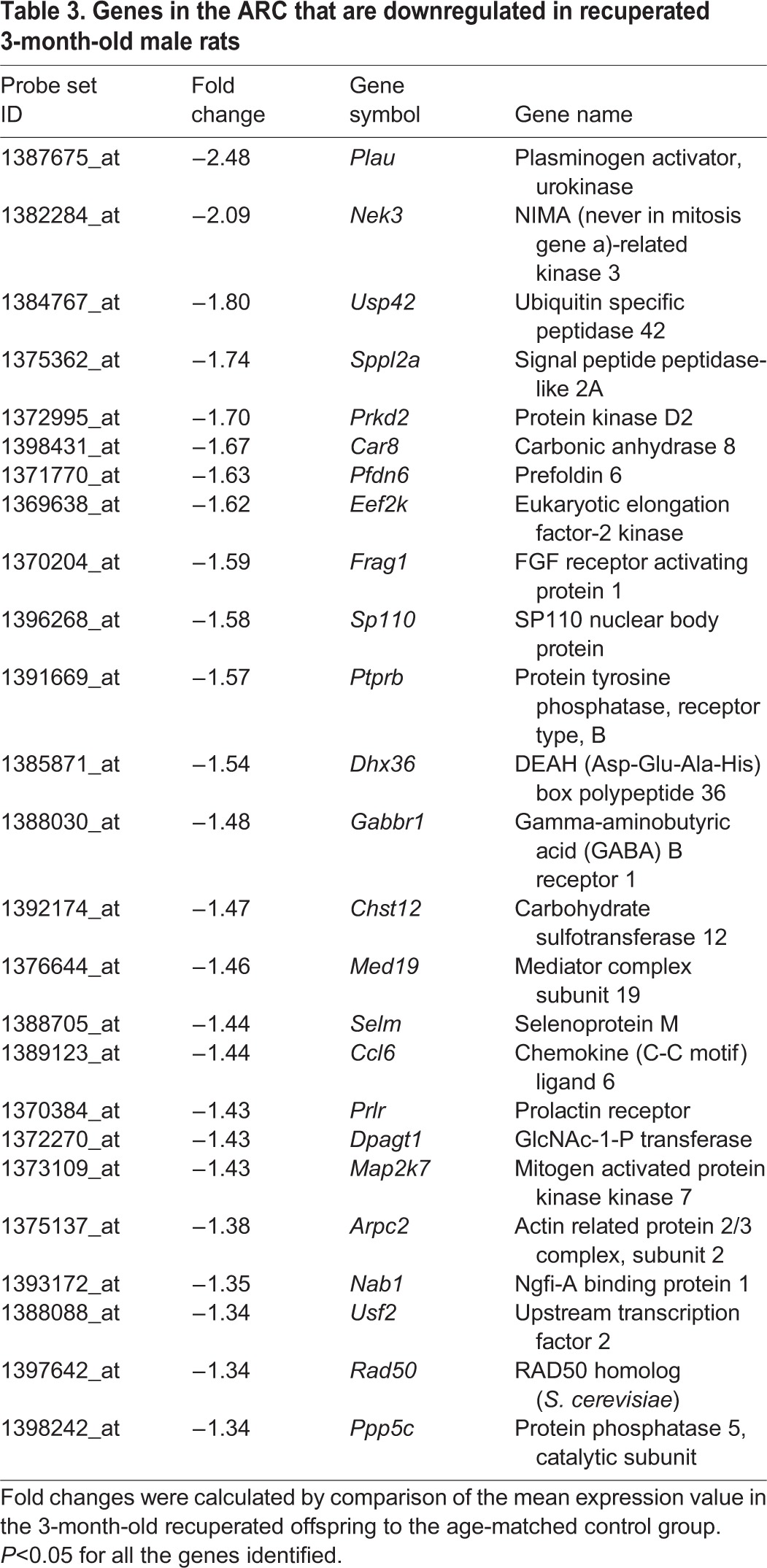
**Genes in the ARC that are downregulated in recuperated 3-month-old male rats**

**Table 4.**
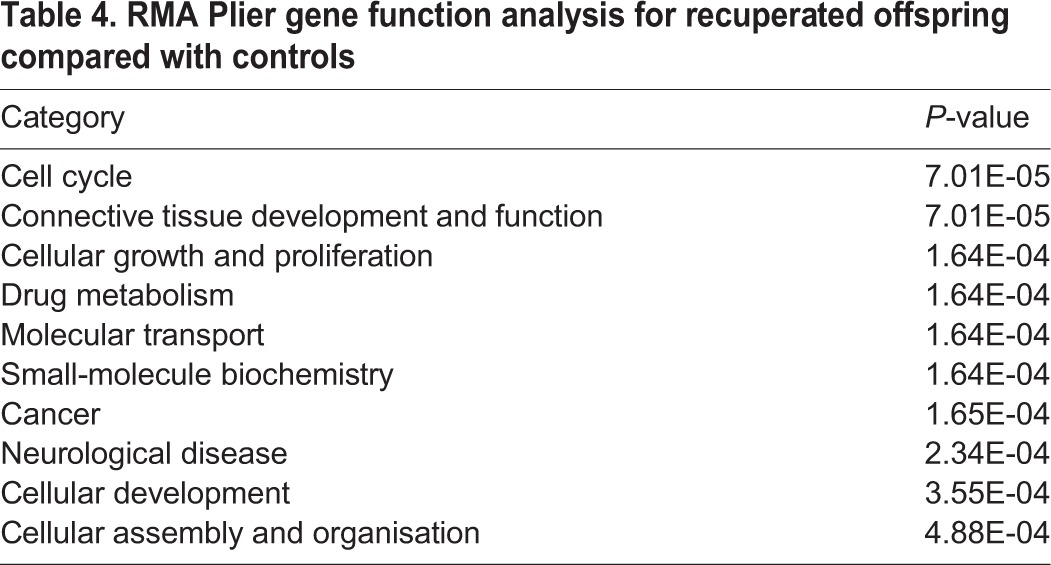
**RMA Plier gene function analysis for recuperated offspring compared with controls**

#### Validation of genes identified by microarray

**Fig. 5.**
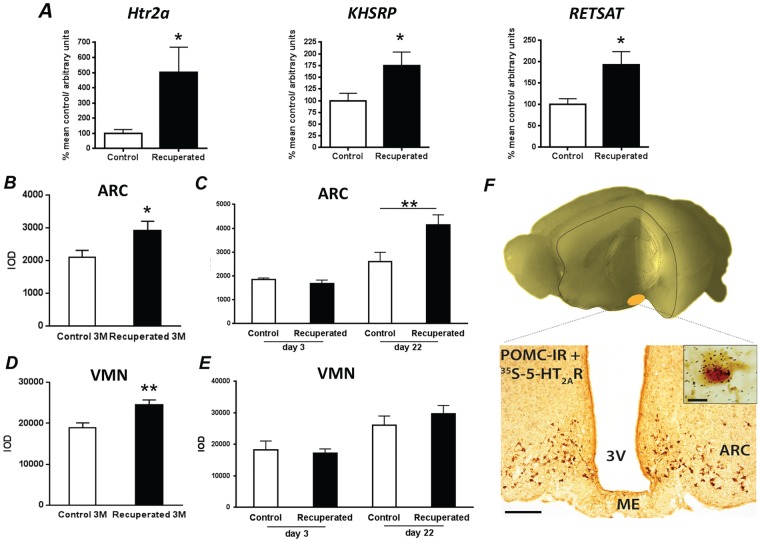
**Validation of the differentially expressed genes identified using microarray approach as being regulated by early nutrition.** (A) Validation of the differentially expressed genes in the ARC of 3-month-old control and recuperated offspring. Analysis was carried out using Taqman qRT-PCR. Gene expression data is normalised to *Ppia.* Data were analysed using a two-tailed unpaired Student's *t*-test. (B) Validation of *Htr2a* mRNA expression in the ARC using ISHH. (C) *Htr2a* mRNA expression in rats at P3 and P22 in the ARC. (D) *Htr2a* mRNA expression in 3-month-old rats in the VMN using ISHH. (E) *Htr2a* mRNA expression in 3 and 22 day old rats in the VMN. (F) Localisation of *Htr2a* on ARC POMC neurons in 3-month-old control rats detected using ISHH for [^35^S]*Htr2a* and chromogenic IHC for α-MSH. Dense black granular staining directly overlapping brown α-MSH cell body and axons indicates the presence of *Htr2a* on POMC neurons. Scale bars: 200 µm for ARC image, 10 µm in the inset. Data were analysed using two-tailed Student's *t*-test in B,D; a two-way ANOVA was used with appropriate Duncan's *post hoc* test for C,E. Values are expressed as means±s.e.m. For A,B,D, *n*=8 per group; for C,E, *n*=6-8 per group, *n* represents number of litters. *P<0.05; **P<0.01. 3V, 3rd ventricle; ME, median eminence.

Out of the seven genes chosen for validation, two genes had very low expression (*Plau* and *Pde4a*). Out of five genes remaining, differential expression of three genes (*Htr2a*, *Khsrp* and *Retsat*) was confirmed by RT-PCR, whereas two genes were not validated (*Car8* and *Eef2k*) (Fig. 5A). As *Htr2a* had the highest fold change in the microarray analysis (Table 2), it was further investigated. The upregulation of *Htr2a* in the ARC of 3-month-old recuperated animals (*P*<0.05) observed using RT-PCR was confirmed by *in situ* hybridisation (*P*<0.05; Fig. 5B).

### Timing and location of increased hypothalamic *Htr2a* expression

A similar pattern of *Htr2a* expression to that seen in 3-month-old animals was observed at P22. *Htr2a* was significantly elevated in the ARC of recuperated rats at weaning (*P*<0.05; Fig. 5C). Expression of *Htr2a* was, however, not altered in neonatal ARC (Fig. 5C) or in E16.5 fetal brains (low-protein, 0.933±0.035; control, 1.002±0.022). As in the ARC, expression of *Htr2a* was also significantly increased in VMN of 3-month-old recuperated animals (*P*<0.05; Fig. 5D,E). However, in contrast to the ARC, no difference was seen in *Htr2a* expression within VMN between the two experimental groups at P22 (Fig. 5E), suggesting that upregulation of *Htr2a* within the VMN in the recuperated group is secondary to the upregulation of this receptor in the ARC. Overall, these findings reveal a change in 5-HT_2A_R receptor expression using an unbiased genome-wide profiling approach and thereby support the importance of maternal diet in inducing changes in the 5-HT system within the offspring hypothalami that may deregulate offspring energy balance.

### Neuronal colocalisation of ARC 5-HT_2A_R and POMC

Within the ARC, 5-HT primarily influences energy balance by 5-HT_2C_R stimulation of POMC neuronal activity (Heisler et al., 2002; Xu et al., 2008; Berglund et al., 2013; Burke et al., 2014). Given that *Htr2a* expression is reduced in rats exposed to a maternal low-protein diet, we hypothesised that *Htr2a* might therefore be upregulated to compensate for this in an effort to normalise energy balance. To determine whether 5-HT_2A_Rs are anatomically positioned to influence the activity of POMC neurons, we visualised co-expression using dual histochemical labelling. We determined that ∼40% of the ARC POMC neurons express *Htr2a* in control rats (Fig. 5F). These data reveal a defined population of neurons within the ARC involved in regulation of body mass that could be influenced by G_q_-protein coupled 5-HT_2A_Rs.

### Effect of 5-HT_2A_R agonism on food intake

**Fig. 6.**
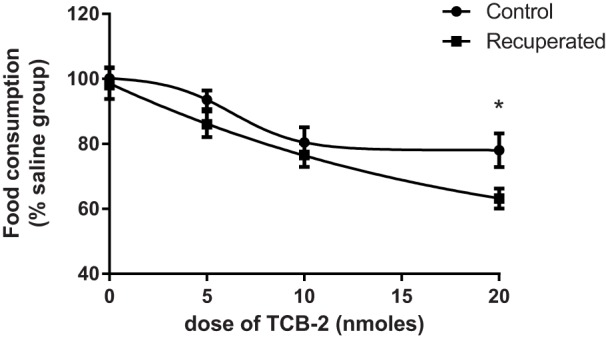
**Effects of maternal protein restriction on food intake following central administration of the 5-HT_2A_R agonist TCB2 in 3-month-old rats.** Food consumption during the 2 h following lights out expressed as a percentage of the saline dosed group. Differences between the groups were analysed using two-way ANOVA (overall effect of TCB2 administration, *P*<0.001; overall effect of early nutrition, *P*<0.05) followed by Bonferroni's multiple comparison test. *n*=13-15 per group, *n* represents number of litters. *P<0.05.

To assess the functional implications of upregulated ARC *Htr2a* expression, we administered the selective 5-HT_2A_R agonist TCB2 [(4-bromo-3,6-dimethoxybenzocyclobuten-1-yl) methylamine hydrobromide] directly into the third ventricle and measured its effects on food intake in adult 3-month-old recuperated and control offspring. We confirmed previous results showing that 5-HT_2A_R agonism significantly suppresses food intake (Fox et al., 2010). TCB2 also significantly decreased food intake in the recuperated offspring, as shown by two-way ANOVA analysis (overall effect of TCB2 administration, *P*<0.001; Fig. 6). However, the dose-response curves between the control and recuperated groups were significantly different (*P*<0.05; Fig. 6). Recuperated offspring were more sensitive than the controls to the centrally administered action of 5-HT_2A_R agonist TCB2 (overall effect of early nutrition, *P*<0.05; Fig. 6). These results reveal that overexpressed 5-HT_2A_Rs in recuperated rats are functional and when pharmacologically stimulated, a greater net effect on appetite is achieved. Extrapolation of these findings to endogenous 5-HT activity, as indicated by the D-fenfluramine results, suggests that the upregulation of 5-HT_2A_R is insufficient to compensate for downregulation of 5-HT_2C_R in the regulation of appetite by 5-HT.

## DISCUSSION

Evidence from epidemiological studies and animal models shows that suboptimal early nutrition affects susceptibility to obesity in later life. We and others have shown that rodents exposed to maternal protein restriction *in utero* and accelerated postnatal growth (recuperated offspring) are more susceptible to development of diet-induced obesity (Ozanne et al., 2004; Bieswal et al., 2006). In the current study, we focused on 3-month-old recuperated rats fed a chow diet that are a similar weight to control offspring. This enabled us to investigate mechanisms mediating the effects of early nutrition on susceptibility to developing diet-induced obesity without the confounding effects of obesity itself. In recent years, leptin has been studied as a key factor involved in programming of obesity risk (Cripps et al., 2009; Bouret et al., 2004). However, we previously showed that programming of increased adiposity is, at least in part, independent of leptin (Cottrell et al., 2011). In the current study, we investigated the effect of a maternal low-protein diet during gestation on a factor directly impacted by reduced dietary protein, 5-HT. We observed that 5-HT levels were increased in the fetal brains and neonatal hypothalami of rats exposed to a low-protein diet *in utero*. Because 5-HT is known as an essential growth and regulatory factor that drives the development and maturation of its own cellular network and related neuronal systems (Pino et al., 2004; Oberlander, 2012), an excess of 5-HT might lead to impaired interneuron migration and abnormal development of the serotonergic system itself and its target regions (Pino et al., 2004; Oberlander, 2012). Here, we suggest that elevated hypothalamic levels of 5-HT in recuperated animals in early life cause developmental and functional alterations that increase susceptibility to obesity later in life.

We observed reduced maternal circulating tryptophan but increased 5-HT in pregnant dams fed a low-protein diet. This could be a consequence of (1) the increased carbohydrate content in the low-protein diet, which was added to balance protein reduction to obtain an isocaloric diet and (2) the dams' increased food intake in both absolute and relative terms (Fernandez-Twinn et al., 2003). Such dietary intervention can affect maternal insulin levels. Low-protein dams were shown to be hyperinsulinaemic at day 14 of pregnancy (Fernandez-Twinn et al., 2003). As circulating tryptophan levels decrease in an insulin-dose-dependent manner, the increased insulin concentration in these dams could contribute to the reduced tryptophan levels (Fernandez-Twinn et al., 2003; Fukagawa et al., 1987). Another possibility is that the dietary intervention has altered the gastrointestinal microbiome of the dam. Diet is the key regulator of both taxa and microbiota production of metabolites (Scott et al., 2013). More than 90% of 5-HT in the body is synthesised in the gut, and the microbiota has been shown to play a crucial role in regulating host 5-HT biosynthesis and 5-HT blood levels (Yano et al., 2015).

Several mechanisms might be involved in mediating the relationship between reduced maternal circulating tryptophan and increased 5-HT in the brains of their unborn offspring. In adult rats, food deprivation leads to an increase in the concentration of circulating free fraction of tryptophan (FFT), the fraction that passes through the blood-brain barrier (Knott and Curzon, 1972; Perez-Cruet et al., 1972). Raised circulating FFT and increases in tryptophan uptake by serotonergic neurons, the activity of tryptophan-5-hydroxylase and synthesis of 5-HT have also been reported in intrauterine growth-restricted (IUGR) rat pups and infants (Kalyanasundaram, 1976; Hernandez et al., 1989; Manjarrez et al., 1996, 1998). Upregulation of FFT in IUGR was attributed to abnormal kinetics of tryptophan binding to albumin (Hernandez-Rodriguez et al., 2009). Reduced plasma levels of leucine, isoleucine and tyrosine, which compete with tryptophan for entry into brain, might also contribute to an increase in the transport of tryptophan across the blood-brain barrier in IUGR pups (Roux and Jahchan, 1974).

In recent years, in both mice and humans, the placenta has emerged as an important source of 5-HT, that can directly impact fetal brain development (Bonnin et al., 2011). In our study, the impact of maternal low-protein diet on 5-HT was more pronounced in the placenta and fetal brain than in the dam. This supports the proposal that the placenta is cannibalised in response to maternal food deprivation to provide nutrients and substrates for the developing hypothalamus (Broad and Keverne, 2011). We observed an association between levels of placental 5-HT and fetal brain 5-HT at E16.5 – the time-point at which 5-HT fibres can be detected in the hypothalamus and both exogenous and endogenous sources of 5-HT contribute to fetal 5-HT synthesis (Aitken and Tork, 1988; Bonnin et al., 2011). Increased 5-HT production within the placenta might contribute to raised 5-HT levels in fetal brains from low-protein dams. Maternal tryptophan levels have also been reported to influence central 5-HT levels in the offspring and maternal peripheral 5-HT proved to be important for offspring neurodevelopment (Bonnin and Levitt, 2011; Cote et al., 2007). This suggests that both maternal and placental tryptophan/5-HT metabolism are part of an important molecular pathway for fetal programming of the 5-HT system and brain development.

Changes in 5-HT bioavailability not only impact formation of hypothalamic circuitry during development but have also been shown to influence appetite and body mass in the adult (Pôrto et al., 2009; Berg et al., 2013; Madden and Zup, 2014). 5-HT primarily regulates energy balance via activity at the G_q_-coupled receptor, 5-HT_2C_R. Here we report that elevated fetal 5-HT could potentially programme decreased levels of hypothalamic 5-HT_2C_R, although direct evidence for this relationship remains to be established. The reduction in 5-HT_2C_R could be an early and permanent developmental consequence of central hyperserotonemia. Reduced 5-HT_2C_R function is known to promote increased appetite and obesity. Specifically, genetic inactivation of *Htr2c*, leads to lifelong hyperphagia and elevated body mass in mice fed standard chow diet (Tecott et al., 1995), whereas pharmacological activation of these receptors reduces food intake and decreases body mass (Heisler et al., 2002; Halford and Harrold, 2012; Burke et al., 2014). 5-HT_2C_Rs are involved in mediating the action of D-fenfluramine, which stimulates the release of 5-HT and inhibits the re-uptake of 5-HT into nerve terminals (Vickers et al., 2001; Trifunovic and Reilly, 2006; Xu et al., 2010). Moreover, the subpopulation of 5-HT_2C_Rs expressed solely with POMC is sufficient to mediate D-fenfluramine and 5-HT_2C_R agonist mCPP appetite suppression (Xu et al., 2008). Following the administration of D-fenfluramine into the third ventricle, we observed impaired sensitivity to 5-HT-stimulated food reduction in 3-month-old recuperated animals. As the functional capacity of 5-HT_2C_R is dependent upon availability of active membrane receptor pools, the impaired response to D-fenfluramine in recuperated animals is probably a consequence of reduced 5-HT_2C_R protein levels in the hypothalamus of these animals.

We used LCM of the ARC combined with microarray analysis to identify differentially expressed genes that could be affected by early hyperserotonemia. We found that *Khsrp* expression was increased in 3-month-old recuperated offspring. *Khsrp*, which encodes KH-type splicing regulatory protein, is involved in the control of mRNA decay (Gherzi et al., 2006; Ruggiero et al., 2007) and plays a key role in the translation of DNA damage signalling to miRNA biogenesis (Trabucchi et al., 2009; Zhang et al., 2011). Although recuperated animals have reduced longevity (Jennings et al., 1999), which is associated with increased oxidative stress and an impaired response to DNA damage (Tarry-Adkins et al., 2008, 2009), the functional significance of the increase in KHSRP in the brain of these animals remains to be determined.

*Htr2a* is another gene that was increased in the 3-month-old recuperated offspring*.* The precise role that *Htr2a* plays in the regulation food intake has not been fully defined*.* *Htr2a* was upregulated in the ARC of diet-induced obese rats (Park et al., 1999), whereas in humans, 5-HT_2A_R correlated positively with BMI and a polymorphism in the gene encoding 5-HT_2A_R has been associated with obesity (Rosmond et al., 2002; Erritzoe et al., 2009; Carr et al., 2013). However, knockout of *Htr2a* in mice does not alter food consumption or gain in body mass when mice are fed laboratory chow (Weisstaub et al., 2006), suggesting the presence of developmental compensatory mechanisms or that body mass gain only occurs when these mice are placed on an obesogenic diet. There is little information regarding the precise neuronal location of 5-HT_2A_R within the ARC. Here, we report that 5-HT_2A_Rs, like 5-HT_2C_Rs (Heisler et al., 2002; Lam et al., 2008), are anatomically positioned to influence the activity of the critical energy balance regulator POMC. The extent to which the activity of POMC neurons can be influenced by action at 5-HT_2A_Rs and whether this mechanism is altered in recuperated offspring as well as direct evidence for linking fetal hyperserotonemia to alteration of 5-HT_2A_R in later life remain to be established.

Because *Htr2a* expression was increased in the ARC of recuperated pups at weaning and in adulthood, but not in brains of fetuses or neonates, the differences in 5-HT_2A_R must be established during the period of catch-up growth, between P3 and P22, much later then the observed differences in 5-HT_2C_R levels. Therefore, upregulation of 5-HT_2A_R might act as a secondary, counter-regulatory response to impaired 5-HT_2C_R signalling. Counter-regulatory mechanisms have been reported among 15 existing 5-HT receptor subtypes as genetic alteration of one specific 5-HT receptor subtype can result in compensatory signalling through another; for example, signalling through 5-HT_1B_R in the absence of 5-HT_2C_R (Heisler and Tecott, 2000; Dalton et al., 2004). It is possible that counter-regulatory upregulation of 5-HT_2A_R in response to impaired signalling through 5-HT_2C_R only emerges when growth-restricted newborn pups are born into and exposed postnatally to an environment of plentiful food. Some effects of early alterations in 5-HT therefore, might only appear in the presence of a particular postnatal environment. This has been shown to be true in a study of 3-month-old infants exposed to a selective serotonin reuptake inhibitor, where altered HPA stress response patterns only became apparent when the method of infant feeding was taken into consideration (Oberlander et al., 2008).

5-HT_2A_R stimulation by administration of agonists produced a greater effect on appetite in recuperated rats, revealing that the overexpressed ARC 5-HT_2A_Rs are functional. As pharmacological stimulation of endogenous 5-HT was less effective in suppressing appetite in recuperated rats, these results suggest that the endogenous circuitry is insufficient to appropriately modulate 5-HT-regulated appetite. However, this change in endogenous programming might be circumvented and the upregulation of the 5-HT_2A_R capitalised upon by pharmacological treatment with a 5-HT_2A_R non-hallucinogenic agonist. The anorectic therapeutic profile could be potentially further improved by combining the 5-HT_2A_R agonist, with 5-HT_2C_R and 5-HT_1B_R agonists, because co-application of 5-HT_2C_R and 5-HT_1B_R agonists produced a significant increase in the activity of POMC in the ARC (Doslikova et al., 2013). Development of a non-hallucinogenic 5-HT_2A_R agonist might seem to be challenging. However, studies showing that the glutamate mGlu2 receptor heterocomplex with 5-HT_2A_R, and not 5-HT_2A_R on its own, acts as a molecular target for the actions of hallucinogenic drugs provide a plausible avenue to explore in the development of therapeutically suitable 5-HT_2A_R agonists (Moreno et al., 2011).

Although we concentrated on studying mechanisms that could mediate the effects of early elevated 5-HT levels on obesity risk in later life, early perturbations in the 5-HT neurotransmitter system are known to have major implications for mental health and behaviour in childhood and adulthood. Increased anxiety-like behaviour in nonhuman primate offspring of dams fed a high-fat diet was associated with perturbations in the 5-HT system during fetal life (Sullivan et al., 2010). Early hyperserotonemia, in particular, has been identified as a potential factor in the pathogenesis of autism spectrum disorder and schizophrenia (Chugani, 2004; Madden and Zup, 2014). In addition, both 5-HT_2A_R and 5-HT_2C_R, which are expressed throughout the brain, have been implicated in the pathophysiology of psychiatric disorders (Abramowski et al., 1995; Hoyer et al., 1986; Heisler et al., 2007; Mestre et al., 2013; Lyddon et al., 2013). 5-HT_2C_R, for example, was shown to play a role in addiction and reward behaviour by modulating dopamine transmission within the mesolimbocortical dopaminergic system (Katsidoni et al., 2011). Dysregulation of the reward-related neurotransmitter systems and behaviours could also contribute to the increased susceptibility to obesity in the recuperated offspring (Grissom et al., 2014).

In summary, we report that elevated 5-HT in fetuses exposed to maternal protein restriction, might underlie a permanent reduction in 5-HT_2C_R expression and function and secondary counter-regulatory upregulation of 5-HT_2A_R and increased sensitivity to 5-HT_2A_R agonists. Thus, our results not only identify a molecular mechanism through which maternal diet might impair offspring energy balance, but also point to a promising pharmacological strategy with 5-HT_2A_R agonist medication to correct this impairment.

## MATERIALS AND METHODS

### Experimental groups and tissue collection

All procedures involving animals were conducted in accordance with the University of Cambridge and the University of Buckingham project licences under the UK Home Office Animals (Scientific Procedures) Act (1986). The breeding of animals was conducted at both the University of Cambridge and the University of Buckingham. Five cohorts of Wistar rats (*Rattus norvegicus*) were established for the studies. One for E16.5 experimental measures, the second for laser-capture microdissection (LCM) and microarray validations, the third for *in situ* hybridisation, the fourth for intracerebroventricular (i3v) and 5-HT/tryptophan measurements and the fifth for protein and mRNA analysis. Detailed information regarding the diet composition and the set-up of the maternal protein restricted and control dams have been published previously (Cripps et al., 2009; Berends et al., 2013). Briefly, on P3, two experimental groups of offspring were established: controls [offspring of control dams (20% protein, w/v), culled to eight (four males and four females) suckled by control dams] and recuperated [offspring of dams fed a low-protein diet (8% protein, w/v) during pregnancy, but nursed by control dams, culled to four males to maximise the plane of nutrition]. The animals were allocated to experimental groups at random. Whole heads were collected from fetuses at E16.5 of pregnancy and from male pups at P3. In addition, hypothalami were dissected from brains of male offspring at P3. The body weight of the remaining pups was recorded at P7, P14 and P21. Following weaning at day 22, one male per litter was culled by a rising concentration of CO_2_ and the brain was dissected. After weaning, the remaining males were fed standard laboratory chow and body mass and food intake were recorded weekly. At 3 months, males were culled and brains were collected. All the dissected brains and heads were frozen on powdered dry ice and were stored at −80°C until further processing. Amniotic fluid and placentas were harvested at E16.5 and serum was prepared from the dams and male pups at P3, weaning and 3 months, and stored at −80°C prior to the measurement of 5HT and tryptophan levels.

### 5-HT and tryptophan assay

Whole brains, placentas, amniotic fluid and serum were used for measurement of 5-HT (DRG International) and tryptophan (Abnova) by ELISA, following the manufacturer's recommendation for sample preparation, acylation (5-HT) and derivatisation (tryptophan). For the measurement of 5-HT in tissue, hypothalamic blocks were excised from frozen brains and extracted as described (Huang et al., 2012). Briefly, samples were homogenised and deproteinised for 30 min in 0.2 N perchloric acid solution containing 7.9 mM Na_2_S_2_O_5_ and 1.3 mM disodium ethyleneamine-tetra-acetic acid. The homogenate was centrifuged at 10,000 ***g*** for 10 min at 4°C and the supernatant assayed for 5-HT.

### Feeding studies

Animals were cannulated as described previously (Stocker et al., 2012). Briefly, a cannula was inserted into the third ventricle under a gaseous anaesthetic (isofluorane: Isoba, Schering-Plough Animal Health) using coordinates from the stereotactic rat brain atlas (Paxinos and Watson, 1998). Its position was verified by a positive drinking response over 15 min to angiotensin II (20 µg ml^−1^ in 2.5 µl). For measurements of acute effects of D-fenfluramine or 5-HT_2A_R agonism on food intake, 3-month-old rats were individually housed, fasted for 4 h, dosed at the beginning of the 12 h dark period and re-fed. Peptide (250 nmol) was given in 2.5 µl saline. Animals were dosed using a Latin square design. Doses were separated by at least 4 days and normal feeding behaviour and body mass was restored prior to administration of the next dose. The specificity and doses of the D-fenfluramine (Tocris Bioscience), or the high-affinity 5-HT_2A_R receptor agonist TCB2 (Tocris Bioscience) were based on published data (Vickers et al., 1999, 2001; Trifunovic and Reilly, 2006; McLean et al., 2006; Xu et al., 2010; Fox et al., 2010) and doses were optimised.

### Western blotting

Hypothalami were dissected from frozen brains according to landmarks: anterior to the optic chiasma, posterior to the mammillary bodies, lateral at the hypothalamic sulcus and superior to the anterior commissure. Dissected hypothalami from P3 and 3-month-old male offspring were homogenised in TK lysis buffer and western blotting analysis was carried out as previously described (Martin-Gronert et al., 2008). Anti-goat primary antibody against 5-HT_2C_R purchased from Santa Cruz Biotechnology (sc-15081, lot D1114) was used at 1:200 dilution. The antibody was validated in two previous studies (Bubar et al., 2005; Anastasio et al., 2010). Anti-goat horseradish peroxidase-conjugated secondary antibody (Jackson ImmunoResearch) was used at 1:10,000 dilution.

### Laser-capture microdissection, RNA isolation and analysis

Hypothalamic sections of the ARC were prepared on a cryostat at 14 µm thickness from approximately −4.52 to −2.30 mm relative to Bregma (Paxinos and Watson, 1998). Sections were collected onto RNase-free membrane-coated slides (PALM Microlaser Technologies) that had been baked at 200°C for 4 h and UV cross-linked for 30 min. Within 24 h of sectioning, sections were placed for 30 s each time in 95%, 75% and 50% ethanol for rehydration. Sections were stained with 1% Cresyl Violet stain (Ambion) for 1 min, dehydrated in graded ethanol concentrations (50%, 75% and twice in 100% for 30 s each time), placed in HistoClear (National Diagnostics) for 5 min and air dried. LCM was performed using a PALM MicrolaserSystem (Fig. 4A). Following microdissection, the captured cells were kept in RNAlater (Ambion). Total RNA was isolated from LCM samples using the RNAqueous Micro RNA extraction kit (Ambion) in accordance with the manufacturer's protocol. The quality and quantity of the RNA samples was determined using Agilent BioAnalyzer PicoChips (Agilent Technologies). Total RNA was isolated from E16.5 fetal heads and neonatal and adult (3 months of age) hypothalami as previously described (Zaibi et al., 2010) and analysed using a NanoDrop ND1000 (Thermo Fisher Scientific).

### RNA amplification

An ovation Pico RNA Amplification System (Nugen Technologies) was used for the amplification of RNA destined for microarray analysis. RNA amplification of LCM ARC samples used to validate genes identified by microarray analysis was performed using a MegaScript T7 Amplification Kit (Ambion) in combination with the GeneChip sample CleanUp Module kit (Affymetrix). The use of a different method of RNA amplification enhanced the validation of the microarray data.

### Microarray hybridisation

The amplified RNA was used for gene expression profiling on Affymetrix Rat Genome 230 2.0 Arrays (Affymetrix) using the Affymetrix GeneChip protocol to fragment and label the target, ready for hybridisation to the arrays (Affymetrix 2004). GeneChip sequences were selected from GenBank, dbEST and RefSeq and the sequence clusters created using UniGene were then further refined by comparison with the publicly available assembly of the rat genome. Microarray hybridisation was carried out by Molecular Biology Services at the University of Warwick, using *n*=6 chips per group.

### Microarray analysis and selection of the genes for validation

Raw image data files were converted to CEL and pivot files using Affymetrix GeneChip Operating Software. All downstream analysis of microarray data was performed using GeneSpring GX 12.0 (Agilent). The CEL files were used for the RMA (Irizarry et al., 2003) and GC-RMA (Wu et al., 2004) analyses, whereas the pivot files were used for GCOS analysis. After importing the data, each chip was normalised to the 50th centile of the measurement taken from that chip and all gene expression data reported as a fold-change from the control state. Genes were considered to be up- or downregulated if the 1.3-fold threshold was reached and *P*<0.05. Only genes that met the above criteria using three different algorithms: GCOS, RMA and GCRMA were taken forward for additional study. The further selection of genes for validation was based on the function of the gene and the availability of suitable primers for validation. Functional analysis was performed using Ingenuity Pathway Analysis (Ingenuity Systems).

### Validation of microarray data using Taqman RT-PCR

Validation of the microarray data was carried out using Micro Fluidic Cards (Applied Biosystems) in accordance with the manufacturer's protocol. The reactions were performed in duplicate for each sample using an ABI 7900HT (Applied Biosystems). A standard curve was constructed for each gene using a serial dilution of pooled cDNA from all LCM ARC samples. The mean C_T_ values of the experimental samples were then used to calculate the relative expression for each sample. The data was normalised to *Ppia* (cyclophilin) expression, which did not change between maternal treatment groups. Real-time PCR (StepOne, Applied Biosystems) was carried out using Assay-on-Demand pre-designed primer and probe sets (Applied Biosystems). Data were analysed using the comparative ΔC_T_ method, comparing recuperated animals with controls. All procedures were carried out in accordance with the manufacturer's recommendation.

### *In situ* hybridisation histochemistry

Coronal sections (20 µm) of frozen hypothalamic ARC, obtained using a cryostat, were thaw-mounted onto poly-L-lysine slides (Polysine, Menzel Glaser, Braunschweig, Germany). Ten sets of slides per animal were serially collected, with the first set of 10 beginning at approximately −4.52 and ending at −2.12 mm, relative to Bregma, according to the atlas of the rat brain (Paxinos and Watson, 1998). For the sectioning of neonatal rat brains, whole heads were used and the sectioning was guided by the neonatal rat brain atlas (Ramachandra and Subramanian, 2011). Slides were stored at −80°C until use.

A [^35^S]-radiolabelled riboprobe targeting nucleotides 1700-1910 of the rat *Htr2a* mRNA transcript was generated by PCR from whole rat brain cDNA. The 210 bp fragment was cloned into pCR-TOPO4 (Life Technologies). For antisense probe generation, *Pst*I linearised recombinant plasmid was subjected to *in vitro* transcription using T7 polymerase in the presence of ^35^S-labelled UTP, as per the manufacturer's instructions (Ambion). The ISHH procedure used has been described in detail previously (Alon et al., 2009; Garfield et al., 2012). Autoradiographic images were quantified using Image Proplus software (Media Cybernetics). Standard curves were generated from ^14^C autoradiographic microscales (Amersham) and integrated optical density (IOD) and area of the hybridisation signal were measured. For each animal, 3-5 sections of ARC (−4.52 mm to −2.12 mm from Bregma) and 2-3 sections for VMN (−3.60 mm to −2.12 mm from Bregma) were analysed. Average ARC and VMH *Htr2a* mRNA expression was calculated.

### Dual-label ISHH and immunohistochemistry

To assess colocalisation of 5-HT_2A_R and POMC, brains taken from control rats, were first processed for detection of *Htr2a* mRNA by ISHH, as described above. Following this, the tissue was washed in PBS before commencement of the IHC protocol to label α-melanocyte-stimulating hormone (α-MSH) protein using procedures previously described (Alon et al., 2009; Garfield et al., 2012). Briefly, sections were incubated in 0.3% H_2_O_2_ in PBS, then rinsed in PBS, blocked in 0.5% BSA/0.5% Triton X-100 in PBS and left in blocking buffer containing rabbit α-MSH antibody (1/10,000; Chemicon, Millipore, MA, USA) overnight. Tissue was then washed in PBS and a biotinylated donkey anti-rabbit secondary antibody (Vector Laboratories) was applied at 1:1000 in blocking buffer. Sections were then washed in PBS, incubated in VectaStain ABC reagent, and following this, chromogenic detection was conducted using 3,3′-diaminobenzidine (DAB) reagent (Vector Laboratories). Sections were mounted onto superfrost slides, dried, then dipped in photographic emulsion (Kodak) and stored at 4°C for 2 weeks before being developed using Kodak developer and fixer. Double-labelled cells were recorded if α-MSH-immunoreactive (IR) cell bodies were overlaid with a [^35^S]*Htr2a* signal greater than 3× background.

### Statistical analysis

Two-tailed unpaired Student's *t*-test was used for statistical analysis and the data are presented as means±s.e.m. unless otherwise stated. Fractional growth rates were calculated using formula: fractional growth rate=(current−starting mass)/(period×starting mass). For microarray data *P*-values were calculated using a two-tailed *t*-test. All data were analysed using GraphPad Prism. Number (*n*) refers to number of litters used. *P*<0.05 was considered statistically significant.

## Data Availability

Data have been deposited in Gene Expression Omnibus (accession number GSE76012) at http://www.ncbi.nlm.nih.gov/geo/query/acc.cgi?acc=GSE76012.
